# Deer antler stem cell-derived exosomes: a regenerative medicine powerhouse from nature’s own repair kit

**DOI:** 10.3389/fcell.2026.1672234

**Published:** 2026-02-02

**Authors:** Xidong Liu

**Affiliations:** School of Biological and Pharmaceutical Engineering, Jilin Agricultural Science and Technology College, Jilin, China

**Keywords:** cell differentiation, deer antler stem cells, exosomes, immunomodulation, regeneration

## Abstract

Exosomes are essential mediators of intercellular communication and, as such, have attracted considerable interest in regenerative medicine. Aantler stem cells (ASCs) have become the central candidate in the field of the periodic regeneration of the deer antlers-the only mammalian organ that can entirely regenerate; they have been found to inherit regenerative characteristics of their parent cells, combined with a low immunogenicity. This is a systematic review of the research advancements on ASC-Exos, including the following main aspects: At the technical level, it describes the isolation techniques (ultracentrifugation, size exclusion chromatography, immunoaffinity capture) and their principles, advantages and disadvantages and their identification methods: TEM (cup-shaped/spherical morphology), DLS (size distribution), Western blot (markers: CD63, CD81, TSG101); In the functional and mechanistic levels, through cargos (proteins, mRNAs, let-7a/let-7b), ASC-Exos enhance fibroblast proliferation/migration (CCK-8, Transwell), osteogenic genes expression (Runx2, Osterix) to differentiate into osteoblasts as well as controlling macrophage polarization (reduce TNF-α, IL-6; enhance IL-10); At the translational application level, ASC-Exos have been shown to be effective in bone repair (rat femoral defect, micro-CT/histology), cartilage protection (alleviate osteoarthritis), wound healing (mouse full-thickness wounds, angiogenesis), pulmonary fibrosis (inhibit CCL7-mediated monocyte-macrophage recruitment), tumor immunotherapy (engineered M2Pep/poly (I: C) ASC-Exos + PD-L1 antibodies suppress tumor growth/metastasis), treatment of neurodegenerative diseases, anti-aging and intervention in age-related diseases and treatment of metabolic disorders. Moreover, this review reveals the challenges in the contemporary research that are of critical importance: optimization of large-scale production and purification of ASC-Exos to provide uniformity in the clinical use; full clarification of the molecular processes that underlie ASC-Exos-mediated effects (e.g., metabolic reprogramming control in tumors); and the absence of detailed preclinical and clinical data on the long-term safety and efficacy. Finally, the review would serve as a valuable resource to developing fundamental research in the field of ASC-Exos and increasing the pace of its clinical application, especially when used together in conjunction with more sophisticated methods of drug delivery and tissue regeneration, to achieve new prospects in the treatment of incurable diseases and repair tissue in regenerative medicine.

## 1 Introduction

Deer antler velvet is the only mammalian organ capable of complete, periodic regeneration and therefore provides an unparalleled model for studying the molecular processes underlying large-scale organ regeneration in mammals (Goss, 1995; Li et al., 2009; Li et al., 2014). The key feature of this regenerative process is a set of pluripotent stem cell (ASC) (Clark et al., 2006; Li et al., 2002; Price et al., 1994) situated in the germinal layer at the base of the deer antler velvet. These ASCs not only exhibit the potential for multidirectional differentiation across the mesoderm and ectoderm, being able to differentiate into various cell lineages including osteocytes, chondrocytes, nerve cells, and vascular cells, but also can meticulously regulate the regeneration of the entire large - scale tissue within an annual regeneration cycle of 4–6 months (Wang et al., 2019). This unique regenerative property renders ASCs a naturally privileged cell source for investigating regenerative medicine strategies, and their paracrine mediators have become the targets of intense research.

Exosomes, nanoscale extracellular vesicles (30–150 nm in diameter), protein-loaded, mRNA-loaded, miRNA-loaded, and bioactive lipid-loaded exosomes have been highlighted in the past few years as fundamental intercellular communication mediators and paracrine-regulated signals. (Thery et al., 2018). In contrast to other exosomes produced by stem cells (like human mesenchymal ones), ASC - derived exosomes (ASC - Exos) have the regenerative signature of their parent cells (Hao et al., 2025; Qin et al., 2023; Zhou et al., 2024), and can thus be considered as having inherent advantages that make them stand out in the realm of regenerative medicine. To begin with, they are lowly immunogenic, which reduces the likelihood of immunotherapy after *in vivo* delivery, an essential requirement for clinical translation (Hao et al., 2025; Zhou et al., 2024). Second, the regenerative and immunomodulatory factors that are highly -specific to the high - efficiency tissue repair process in deer antler are enriched in the molecular cargo of ASC - Exos, allowing the cell proliferation, differentiation, and microenvironmental homeostasis to be targeted more precisely than other exosomes (Hao et al., 2025; Meng et al., 2024; Stoorvogel et al., 2002). Third, as a natural product, ASC-Exos possess biocompatibility and biosafety, based on the original use of deer antler, a natural resource in traditional medicine, which provides ASC-Exos with a strong starting point for the development of therapeutic agents (Hao et al., 2025; Zhou et al., 2024).

These distinctive strengths address major bottlenecks in regenerative medicine, including the low efficacy of current treatment options, the high likelihood of immune rejection, and the poor biocompatibility of artificial implants. Thus, ASC-Exos are emerging as a research focus area with significant potential for breakthroughs in the treatment of refractory diseases and in tissue repair. Growing evidence supports the view that ASC-Exos are essential for tissue repair, immune regulation, and angiogenesis via the paracrine pathway (Liu et al., 2023), and show great promise in bone defect repair, nerve regeneration, and anti-aging studies (Yang et al., 2024). Against this background, this review critically summarizes advances in ASC-Exos, their isolation and identification methods, biological roles, and translational studies. It also addresses current issues, to offer a systematic approach to the development of basic research and the expedited clinical translation of ASC-Exos.

## 2 Research progress of exosomes derived from deer antler stem cells

### 2.1 Isolation and identification of exosomes derived from deer antler stem cells

#### 2.1.1 Isolation methods

The isolation of exosomes is a demanding requirement in order to investigate their biological processes and translation usage, since various techniques of isolating exosomes can influence the quantity, purity, and composition of exosomes as well as the future biological functionality of exosomes. Three commonly used ASC-Exos isolation methods are detailed below and possible compositional and functional differences are discussed, as well as their implications in application.

##### 2.1.1.1 Ultracentrifugation

Widely recognized as the gold standard, ultracentrifugation is as a prevalent method for isolating ASC-Exos supernatants. This approach relies on a sequential series of high-speed centrifugation steps to fractionate exosomes from other supernatant components. Initially, low-speed centrifugation (e.g., 300–500 × g for 10–15 min) is employed to pellet cells and cell debris, effectively clarifying the supernatant. Subsequently, medium-speed centrifugation (10,000–20,000 × g for 30–60 min) is utilized to remove larger vesicles. Finally, exosomes are isolated by ultra-speed centrifugation (100,000–150,000 × g for 70–120 min) (Thery et al., 2006). Despite its effectiveness, this methodology is labor-intensive and time-consuming. Moreover, the intense forces generated during ultra-high-speed centrifugation can potentially damage exosome integrity, compromising the quality of the isolated vesicles.

Compositional and functional differences

A key advantage of this method lies in its capacity to process large-volume samples while achieving relatively high total yields of exosomes, rendering it well-suited for preliminary functional screening that demands substantial starting materials. Nevertheless, the intense mechanical forces induced by ultra-high-speed centrifugation may compromise the integrity of exosomal membranes. This can lead to conformational alterations or shedding of membrane-bound proteins (e.g., CD63, CD81) and potential leakage of partially water-soluble microRNAs (miRNAs), such as let-7a and let-7b (Corona et al., 2023).

Recent studies have demonstrated that ASC-Exos isolated via ultracentrifugation exhibit approximately 15%–20% lower levels of osteogenesis-related proteins (e.g., Runx2 regulatory factors) compared to those obtained through alternative methods. As always, their activity in stimulating osteoblast differentiation is also reduced to a comparable extent during *in vitro* osteogenesis (Zuo et al., 2024). Additionally, ultracentrifugation can separate protein aggregates and small vesicles with similar sedimentation coefficients, yielding moderate exosome preparations from the isolated vesicles. Indicatively, in wound-healing system models, there are inconsistent results in fibroblast-migration assays because of contaminants that can affect particular exosome-target cell interactions (Zhang et al., 2023b).

Implications for applications

A key application of ultracentrifugation is large-scale preliminary studies, e.g., initial assessment of the overall regenerative potential of ASC-Exos or high-throughput screening of functional cargos. Nevertheless, when high-purity, intact-structure exosomes are required, as in mechanism-specific studies or *in vivo* targeted therapy, this approach should be supplemented with purification steps (e.g., size-exclusion chromatography) to improve sample quality. Future directions for optimization could include adjustments to centrifugation parameters (e.g., reduced centrifugal force or prolonged centrifugation time) or the use of protective agents to mitigate membrane damage while maintaining yield and functional integrity.

#### 2.1.1.2 Size exclusion chromatography

It is a technique used to separate exosomes and deer antler stem cell culture supernatant through a column filled with porous beads, in which the supernatant is passed through. These nanovesicles will be separated at a different time point, as their size alone (exosomes measure 30–150 nm) enables their separation from other macromolecules (e.g., proteins and protein aggregates) and smaller vesicles. One of the most important benefits of this technique is the possibility to obtain exosome preparations of high purity and low structural damage (Mathivanan et al., 2010). However, like ultracentrifugation, size-exclusion chromatography is time-consuming and has low sample throughput, which may be problematic in extensive studies.

Compositional and functional differences

Exosomal membranes and the stability of their internal cargos can be preserved, since size-exclusion chromatography (SEC) is a gentle separation technique. Proteomic and transcriptomic assays have demonstrated that the protein/microRNA (miRNA) composition of adipose-derived stem cell exosomes (ASC-Exos) obtained in this manner is more homogeneous. Interestingly, the levels of key regenerative factors, such as let-7a/let-7b and osteogenic growth factors, in them are much higher than those obtained by ultracentrifugation (Meng et al., 2024).

Experiments on cartilage repair revealed that ASC-Exos isolated by SEC were 30% more syngeneic in promoting chondrocyte proliferation and differentiation, as well as in promoting type II collagen synthesis, compared with the purified samples isolated by ultracentrifugation. This is because exosomal membranes are not disrupted, thereby increasing cargo delivery to target cells (Lei et al., 2022). Additionally, SEC-isolated exosomes exhibit fewer general biological activities (e.g., immunomodulation) because they are not contaminated to the same extent. Macrophage polarization studies also indicate that these exosomes decrease tumor necrosis factor-α (TNF-α) secretion and enhance interleukin-10 (IL-10) expression, without affecting foreign protein recognition (Zhang et al., 2023a).

Implications for applications

SEC is well-suited for preclinical efficacy and mechanism-oriented studies, where high exosome yield and purity are required. The reliability and reproducibility of the study results can be ensured by the stability of the cargo content of SEC-isolated exosomes, as an example of such a study where the role of specific miRNAs (e.g., let-7b) in the treatment of pulmonary fibrosis is studied (Zhang et al., 2023a). Nevertheless, the approach has internal limitations, such as low sample throughput and time-consuming processes, which preclude its applicability to large-scale production. A future enhancement pathway is to develop high-throughput SEC columns or integrate SEC with microfluidic technology to improve separation efficiency while maintaining exosome quality.

#### 2.1.1.3 Immunoaffinity capture method

The technique takes advantage of the specific bacterial interaction between antibodies and exosome-specific surface markers, e.g., CD63 and CD81. Immunoaffinity magnetic beads complexed with the respective antibodies are used to selectively isolate exosomes from the deer antler stem cell culture supernatant, yielding well-purified exosome preparations. The benefit of using this technique is that extremely purified exosome preparations are obtained. Nevertheless, it is characterized by relatively high costs, as it requires specific antibodies and magnetic beads. Additionally, the exosomes obtained through this technique may have low yields, which can limit their use in large-scale studies and clinical practice (van Niel et al., 2018).

Compositional and functional differences

Of the three approaches, immunoaffinity capture is the most pure. It thus allows purification of exosomes with homogeneous expression of surface markers and minimal contamination by other vesicles or proteins. This extraordinary specificity is ensured by the purity of functional studies, such as the use of engineered ASC-Exos [i.e., M2Pep-Exo (pIC)] identified through this purification method, which targets M2 macrophages explicitly and avoids off-target effects caused by impurities (Yang et al., 2024).

The capture process can, however, modify the conformation of surface markers via antibody binding, which may block the interaction and identification of exosomes and target cell membranes. Moreover, the technique has the disadvantage of low yields, as it isolates exosomes that express only the target markers and does not isolate other functionally relevant subpopulations. For example, some ASC-Exos that lack the canonical markers (e.g., CD63/CD81) but carry critical regenerative cargos can be eliminated during isolation, which reduces total functional activity in individual tissue repair models (Corona et al., 2023).

Implications for applications

Immunoaffinity capture is particularly well suited to studies that involve a specific exosome subpopulation, validate a particular functional role (e.g., the role of CD63-positive ASC-Exos in osteogenesis), or investigate the immunomodulatory impact of marker-specific exosomes. It is also highly pure, which makes it suitable for the preparation of exosomes for clinical use; however, the high cost of antibodies and magnetic beads limits large-scale use. The next step could be the development of multi-target antibody conjugates to integrate a broader range of functional exosome subpopulations, or the optimization of bead-recycling technology to reduce production costs.

#### 2.1.2 Identification methods

##### 2.1.2.1 Transmission electron microscopy (TEM)

Transmission electron microscopy (TEM) is a standard and trustworthy method of examining the morphology of exosomes. Under TEM, ASC-Exos are mainly in the form of cups or a sphere, and the diameter of the ASC-Exos is usually between 30 and 150 nm (Corona et al., 2023). This morphological feature of exosomes is readily identifiable and typically reflects their characteristic shape and size.

##### 2.1.2.2 Size analysis (DLS and NTA)

Dynamic light scattering (DLS) is a beneficial analytical methodology used in the identification of the size of exosomes available in solution. Exosome size can also be measured using DLS because it will identify the differences in the light reflected off of the vesicles The DLS-derived data on size that fall within the exosome size range (30–150 nm) provide further support for the conclusion that the size-isolated particles are exosomes. It is a highly quantitative method that can supplement morphological data and enhance the accuracy of exosome detection. Additionally, fluorescent nanoparticle tracking analysis (NTA) is used todetermine the size and phenotype of cellular vesicles in the short term (Dragovic et al., 2011). This system involves observing vesicles using an optical microscope in the light-scattering mode. Videos are recorded, and the NTA software is used to track the Brownian motion of individual vesicles and to calculate their size and concentration. It has been shown that NTA can detect cellular vesicles as small as approximately 50 nm. It is more sensitive than conventional flow cytometry (with a lower limit of approximately 300 nm). Using NTA and fluorescence in combination, it has now been established that vesicles can be labeled with quantum dotsto which antibodies are conjugated, thereby enabling identification of their phenotype.

##### 2.1.2.3 Marker protein detection

Western blot and flow cytometry are two widely employed methods for detecting exosome - specific marker proteins. These methods were used to determine the protein expression levels of CD63, CD81, and TSG101 in the isolated vesicles. The identification of these markers strongly indicates that the isolated vesicles are exosomes, as these proteins are uniquely associated with exosome biogenesis and composition. Western blotting can be used for detailed protein profiling and quantification, whereas flow cytometry enables high - throughput analysis to rapidly screen and characterize exosome populations (Thery et al., 2018).

### 2.2 Biological function of deer antler stem cell-derived exosomes

**FIGURE 1:**
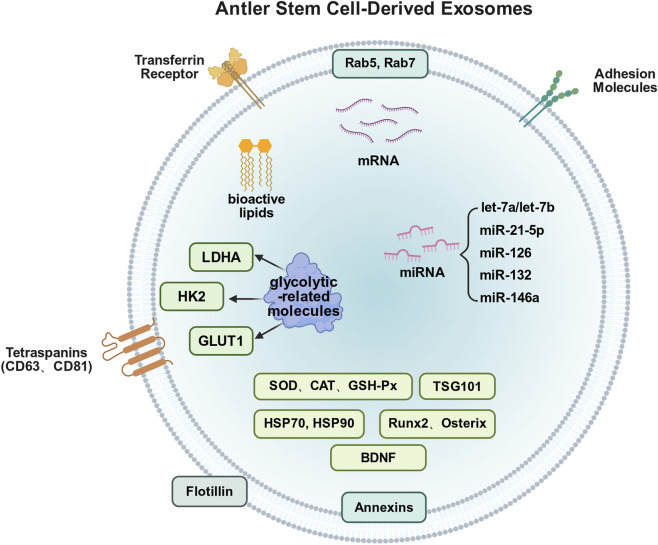
Schematic Diagram of Core Components of Antler Stem Cell-Derived Exosomes (ASC-Exos). This figure presents the core constituent components of ASC-Exos, which are composed of multiple categories of bioactive key molecules, specifically: Surface markers: These include tetraspanins (CD63, CD81), Transferrin Receptor, Rab5/Rab7, adhesion molecules, Flotillin, Annexins, TSG101, and Heat Shock Proteins (HSP70, HSP90). These molecules are unique to exosomes and serve as important markers for their identification and verification. Nucleic acids: ASC-Exos contain mRNA and a variety of functional microRNAs (miRNAs), such as let-7a/let-7b, miR-21-5p, miR-126, miR-132, and miR-146a. These nucleic acid molecules participate in regulating critical biological processes of target cells, including cell proliferation, differentiation, and metabolic regulation. Proteins and lipids: The components also include bioactive lipids, antioxidant enzymes (SOD, CAT, GSH-Px), osteogenesis-related transcription factors (Runx2, Osterix), Brain-Derived Neurotrophic Factor (BDNF), and glycolysis-related molecules (GLUT1, HK2, LDHA). These diverse components work together to form the functional network of ASC-Exos, providing a solid material basis for their biological effects in intercellular communication, tissue repair, immunomodulation, and other physiological and pathological processes.

**FIGURE 2:**
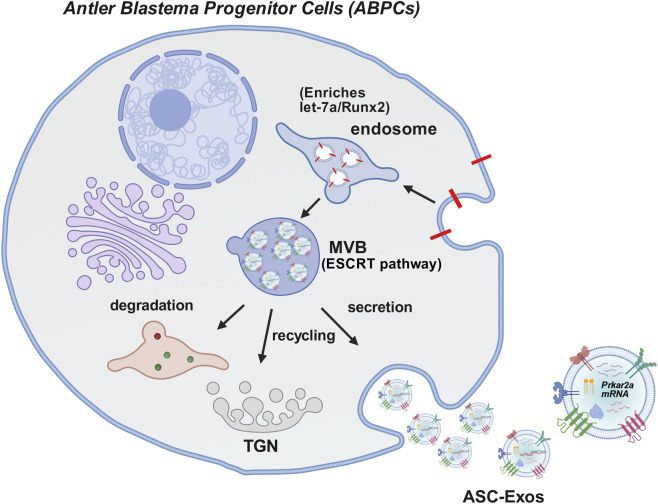
Biogenesis and Secretion of Antler Stem Cell-Derived Exosomes (ASC-Exos) from Antler Blastema Progenitor Cells (ABPCs). This diagram depicts the formation and secretion process of exosomes (ASC-Exos) in Antler Blastema Progenitor Cells (ABPCs), with key steps as follows: Endosome enrichment: Early endosomes within ABPCs selectively gather functional molecules, such as let-7a miRNA and Runx2 protein, laying the foundation for exosome function.MVB formation: Guided by the ESCRT pathway (Endosomal Sorting Complex Required for Transport), early endosomes undergo maturation to form Multivesicular Bodies (MVBs). These MVBs contain intraluminal vesicles that will eventually develop into exosomes. MVB fate: Mature MVBs have three possible developmental directions: Degradation: Fusion with lysosomes to break down internal components; Recycling: Fusion with the Trans-Golgi Network (TGN) for component reuse in cellular processes; Secretion: Fusion with the cell membrane to release intraluminal vesicles into the extracellular environment, which are then defined as ASC-Exos. ASC-Exos content: The secreted ASC-Exos carry a variety of bioactive cargos, including PrKar2a mRNA, functional proteins, and nucleic acids. These cargos serve as messengers for intercellular communication, enabling signal transmission between ABPCs and target cells.

**FIGURE 3:**
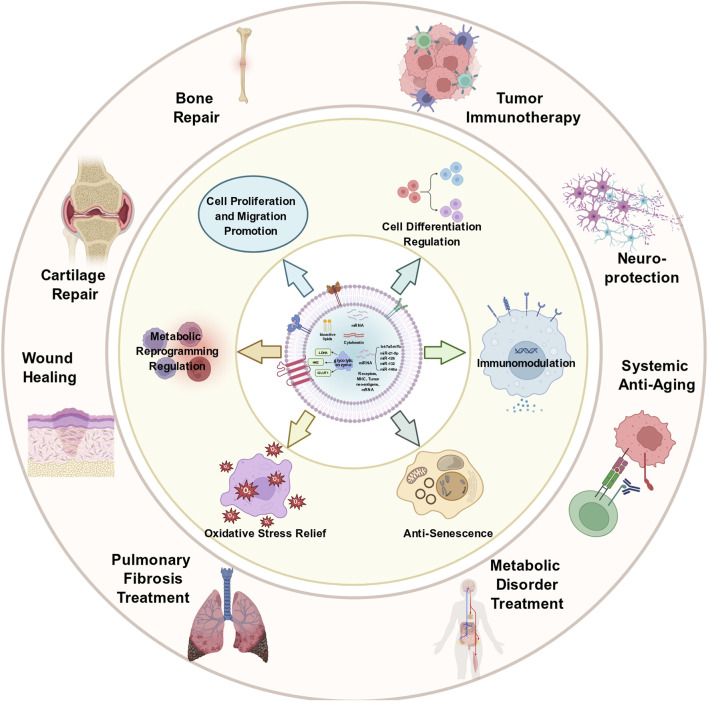
Multifunctional Biological Effects and Therapeutic Applications of Antler Stem Cell-Derived Exosomes (ASC-Exos). This diagram outlines the core regulatory functions of ASC-Exos and their corresponding potential therapeutic application scenarios: Central functional mechanisms: ASC-Exos exert six key regulatory effects on target cells and tissues: Promotion of cell proliferation and migration: Accelerating the proliferation and migration of repair-related cells (e.g., fibroblasts, vascular endothelial cells); Regulation of cell differentiation: Guiding the directional differentiation of stem cells or progenitor cells (e.g., osteogenic, chondrogenic, neurogenic differentiation); Immunomodulation: Regulating the polarization of macrophages, balancing T cell subsets, and inhibiting excessive immune activation; Anti-senescence: Inhibiting cellular senescence and delaying the aging process of tissues; Alleviation of oxidative stress: Enhancing the activity of antioxidant enzymes and reducing oxidative damage to cells; Regulation of metabolic reprogramming: Adjusting the metabolic mode of target cells to adapt to the microenvironment of tissue repair or disease progression. Therapeutic application scenarios: Based on the above multi-faceted functions, ASC-Exos show broad application potential in multiple fields of disease treatment and tissue repair: Tissue repair: Bone defect repair, cartilage damage repair, and skin wound healing (including diabetic wounds); Disease treatment: Treatment of pulmonary fibrosis, regulation of metabolic disorders (e.g., type 2 diabetes, obesity), and tumor immunotherapy; Physiological regulation: Neuroprotection (alleviating neurodegenerative diseases such as Alzheimer’s and Parkinson’s), and systemic anti-aging (improving age-related phenotypes in multiple tissues).

ASC-Exos inherit the regenerative and regulatory properties of parent antler stem cells (ASCs) and exert biological effects through their encapsulated bioactive cargos (proteins, mRNAs, miRNAs), as illustrated in Figures 1–3. Beyond the previously described core functions, their roles in anti-senescence, neuroprotective regulation, and metabolic modulation have been increasingly validated, forming a multi-dimensional functional network.

#### 2.2.1 Promote cell proliferation and migration

Numerous studies have demonstrated that ASC-Exos can significantly promote the proliferation and migration of various cell types, laying a foundation for tissue regeneration.

In - vitro experiments using the CCK - eight assay showed that ASC-Exos treatment increased fibroblast proliferation rates by 30%–50% compared to controls (Zhang et al., 2023b). Transwell migration assays indicated that these exosomes could also enhance the migratory ability of fibroblasts by regulating the epithelial-mesenchymal transition (EMT) pathway, with migration rates doubling within 24 h (Meng et al., 2024). For vascular endothelial cells, ASC-Exos upregulate the expression of migration-related proteins (e.g., VEGF receptor 2) through delivering miR-21-5p, accelerating endothelial cell recruitment to injury sites and promoting neovascularization (Meng et al., 2024).

Mechanistically, this function is closely associated with the activation of downstream signaling pathways (e.g., STAT3, PI3K/Akt) by ASC-Exos-derived growth factors (e.g., bFGF, EGF) and miRNAs, which shorten the cell cycle and enhance cytoskeletal remodeling (Zhang et al., 2023b). The key bioactive cargos mediating these effects (e.g., miR-21-5p) and their corresponding applications are summarized in Table 1.

**TABLE 1:** Three-dimensional correspondence table of ASC-Exos components, core biological functions, and application scenarios. The table summarizes the key bioactive cargos of antler stem cell-derived exosomes, their targeted biological functions, and corresponding translational application directions, with relevant references provided for validation.

Components	Core biological functions	Application functions	Corresponding references
miR-21-5p	Promote proliferation/migration of fibroblasts/vascular endothelial cells	Wound healing	Meng et al., 2024; Zhang et al., 2023b
Runx2 mRNA, Osterix mRNA, let-7a/let-7b	Regulate osteogenic differentiation	Bone repair and anti-osteoporosis	Liu et al., 2020; Hao et al., 2025
ACAN, collagen type II mRNA	Regulate chondrogenic differentiation (promote cartilage ECM synthesis)	Cartilage repair and anti-osteoarthritis	Zhou et al., 2024; Lei et al., 2022
Let-7a/let-7b	Immunomodulation (inhibit monocyte-macrophage recruitment)	Pulmonary fibrosis treatment	Zhang et al., 2023a; Li et al., 2021
M2Pep/poly (I:C) Engineered ASC-Exos	Immunomodulation, metabolic reprogramming, tumor microenvironment remodeling	Tumor immunotherapy	Yang et al. (2024)
Prkar2a mRNA, SOD, BRCA1 (upregulated), p16INK4a/p21CIP1 (downregulated)	Anti-senescence (inhibit cell senescence, promote DNA repair)	Systemic anti-aging	Hao et al., 2025; Lei et al., 2022
SOD, CAT, GSH-Px, miR-126	Alleviate oxidative stress	Pulmonary fibrosis treatment, cartilage repair and anti-osteoarthritis, wound healing	Li et al., 2021; Zhang et al., 2023b
GLUT1, HK2, LDHA	Promote glycolytic metabolism (metabolic reprogramming)	Wound healing, bone repair and anti-osteoporosis	Yang et al., 2024; Liu et al., 2023
BDNF, miR-132, miR-124-3p	Neuroprotection (promote NSC differentiation, inhibit neuronal apoptosis)	Anti-neurodegenerative diseases (AD, PD)	Yang et al. (2024)
miR-146a	Regulate glucose-lipid metabolism	Metabolic disorder treatment (T2D, obesity)	Liu et al., 2023; Li et al., 2023
EVs_ABPC	Anti-senescence, osteogenesis, systemic homeostasis regulation	Systemic anti-aging, bone repair and anti-osteoporosis	Hao et al. (2025)
Deer antler-specific Glycosphingolipids	Signal regulation, stem cell activity maintenance	Cartilage repair, Neuroprotection, bone repair	Zhou et al., 2024; Yang et al., 2024
CD63, CD81, TSG101	Exosome purity/activity verification	All application scenarios	Thery et al., 2018; Corona et al., 2023

#### 2.2.2 Regulation of cell differentiation

Exosomes secreted by deer antler stem cells have the capacity to regulate the differentiation of target cells, particularly in mesodermal and ectodermal lineages, contributing to tissue-specific repair.

Osteogenic differentiation: When co - cultured with osteoblasts, these exosomes can upregulate the expression of osteoblast - related genes, such as Runx2 and Osterix, thereby promoting the differentiation of osteoblasts into mature osteocytes. It may be linked to the effects of microRNAs or proteins present in exosomes, which can alter intracellular signaling pathways involved in osteogenic differentiation (Liu et al., 2020). Details of the core components and their roles in cell differentiation are provided in Table 1.

##### 2.2.2.1 Chondrogenic differentiation

ASC-Exos promote cartilage-specific extracellular matrix (ECM) protein production (type II collagen and proteoglycans) in chondrocyte cultures by loading them with type II collagen mRNA and anti-senescence proteins. This signaling pathway promotes chondrocyte dedifferentiation in response to inflammatory mediators (Lei et al., 2022).

##### 2.2.2.2 Neurogenic differentiation

Early findings indicate that ASC-Exos can induce neural stem cells (NSCs) to differentiate into fully functional neurons and astrocytes. *In vitro* experiments have demonstrated that NSC differentiation can be triggered by neurotrophic factors (e.g., BDNF, GDNF) and by the let-7 family miRNAs secreted by ASC-Exos, leading to a 40% increase in the number of cells expressing the neuron-specific marker β-III tubulin (Yang et al., 2024).

#### 2.2.3 Immunomodulatory function

Exosomes derived from deer antler stem cells may also possess immunomodulatory properties. These exosomes can regulate macrophage activation and cytokine release across a variety of *in vitro* immune cell models.

Macrophage polarization control: ASC-Exos suppress pro - inflammatory cytokines production, including tumor necrosis factor - α (TNF - α) and interleukin - 6 (IL - 6), and increase the synthesis of anti - inflammatory cytokines, such as interleukin - 10 (IL - 10). This means that they can regulate the immune microenvironment (Zhang et al., 2023a). The exosomal proteins (e.g., CD206 ligand) and miRNAs (let-7a/let-7b) are the mediators of this polarization change between M1 and M2 macrophages that prevent the activation of the NF-κB signalling pathway.

##### 2.2.3.1 Regulation of T Cell responses

ASC-derived exosomes (ASC-Exos) support the maintenance of T cell subsets by attenuating the expansion of pro-inflammatory CD4^+^ T cell subsets, such as Th1 and Th17 cells, and by promoting the expansion of regulatory T cells (Tregs). In particular, ASC-Exos enhance the percentage of Tregs in the peripheral blood mononuclear cells (PBMCs) by 30% (Yang et al., 2024). This alteration in T cell polarization suppresses excessive immune activation and prevents immune-mediated tissue damage.

##### 2.2.3.2 Inhibition of dendritic cell (DC) maturation

ASC-Exos also inhibit maturation of the DCs, with the expression of notable markers of maturation, such as CD80, CD86, and MHC class II molecules being downregulated. ASC-Exos can induce local immune tolerance by inhibiting T-cell activation via DCs (Yang et al., 2024).

#### 2.2.4 Anti-senescence and oxidative stress alleviation

ASC-Exos inhibit senescence and minimize oxidative damage in delaying the active life span of repair-associated cells.

Anti-senescence effect: ASC-Exos treatment inhibits the senescence-associated rate of mesenchymal stem cells (MSCs) and fibroblasts by 40%–50% of the levels of senescence-associated β-galactosidase (SA-β-gal) (Lei et al., 2022). The mechanistic pathways involve ASC-Exos transferring Prkar2a mRNA and SOD proteins, which upregulate DNA repair genes (e.g., BRCA1) and downregulate senescence-linked genes (e.g., p16INK4a, p21CIP1) (Hao et al., 2025).

Oxidative stress alleviation: In lung fibroblasts and chondrocytes exposed to reactive oxygen species (ROS), ASC-Exos increase the activity of antioxidant enzymes (SOD, CAT, GSH-Px) by 1.5 to 2 folds, reducing intracellular ROS levels and lipid peroxidation (Li et al., 2021; Zhang et al., 2023a). This is attributed to the transfer of mitochondrial function-regulating miRNAs (e.g., miR-126) that protect mitochondrial integrity and inhibit apoptotic signaling (Li et al., 2021).

#### 2.2.5 Metabolic reprogramming regulation

Recent studies have revealed that ASC-Exos modulate the metabolic phenotype of target cells, enhancing their adaptability to the repair microenvironment.

Glycolytic metabolism promotion: ASC-Exos upregulate the expression of glycolytic-related molecules (GLUT1, HK2, LDHA) in fibroblasts and osteoblasts, shifting cellular metabolism from oxidative phosphorylation to glycolysis (Zhu et al., 2024). This metabolic reprogramming increases ATP production under hypoxic conditions (e.g., wound sites, bone defects), supporting cell proliferation and ECM synthesis.

Lipid metabolism regulation: In tumor-associated macrophages (TAMs), lipid accumulation and fatty acid oxidation are inhibited by engineered ASC-Exos (M2Pep-Exo (pIC)), thereby reversing the immunosuppressive metabolic phenotype of TAMs and improving antigen-presenting capacity (Yang et al., 2024).

### 2.3 Potential applications in disease treatment and tissue repair

The ASC-Exos, owing to their multidimensional biological functions summarized in Figure 3, have proven to be promising therapeutic agents for a wide range of diseases. In addition to the scenarios mentioned earlier, their uses have extended to neurodegenerative diseases, metabolic diseases, and age-related diseases, indicating their utility in regenerative medicine.

#### 2.3.1 Bone repair

Local injection of ASC-Exos can stimulate bone regeneration in animal models of bone defects. These exosomes can attract endogenous stem cells to sites of damage, stimulate their osteogenic differentiation, and remodel the deposition of new bone matrix. To illustrate, a rat model of the femoral defect evaluated by microcomputed tomography (micro - CT) and histological data indicated that both new bone formation and regeneration were higher in the presence of deer antler stem cell - derived exosomes compared to the one treated with saline (Zuo et al., 2024). They can be associated with various clinical indicators, including traumatic bone defects, osteonecrosis of the femoral head, and osteoporosis, among which the advantages of minimal invasiveness and immunogenicity are most relevant.

#### 2.3.2 Cartilage repair

Cartilage diseases, such as osteoarthritis, may also be treated with ASC-Exos. They can also induce chondrocyte proliferation and modulate chondrocytes via inflammatory cytokines. Introduction of a deer antler stem cell - derived exosomes into the *in vitro* animal cultures of osteoarthritis reversed the reduction in type II collagen and proteoglycan expression in the chondrocyte cultures induced by inflammatory cytokines. This indicates that these exosomes can slowthe progression of cartilage degeneration (Lei et al., 2022).

#### 2.3.3 Wound healing

ASC-Exos can accelerate the wound - healing. They have the ability to stimulate the migration and proliferation of keratinocytes and fibroblasts at the wound margin, and also the appearance of new blood vessels within the wound area. Topical deer antler stem cell - derived exosomes as an agent in the treatment of a mouse model of a full-thickness skin wound model significantly reduced the wound - healing period, enhanced the density of the new blood vessels, and enhanced wound repair quality (Meng et al., 2024; Zhang et al., 2023b). For diabetic wounds, ASC-Exos-loaded hydrogels (e.g., CEGA hydrogel) achieve synergistic effects of antibacterial activity, anti-oxidation, and pro-angiogenesis, reducing the infection rate by 60% and promoting collagen fiber remodeling (Zhang et al., 2023b).

#### 2.3.4 Treatment of pulmonary fibrosis

Pulmonary fibrosis (PF) is a chronic and progressive interstitial lung disease, typically characterized by the excessive deposition of the extracellular matrix (mainly type I collagen), which leads to the continuous deterioration of lung function. Currently, treatment options are limited, and their effectiveness is not satisfactory. Zhang et al. (2023a) established a mouse model of PF and found that the administration of deer antler stem cell - derived exosomes (ASC - Exos) could significantly increase the survival rate of PF mice, reduce the degree of pulmonary fibrosis, and decrease collagen deposition and myofibroblast differentiation. Upon in - depth exploration of the mechanism, it was found that ASC - Exos did not inhibit the polarization of M2 macrophages but was related to the inhibition of monocyte - macrophage recruitment. During the development of pulmonary fibrosis, M2 macrophages secrete profibrotic factors, which accelerate the progression of the disease. ASC - Exos indirectly inhibit fibrosis by reducing the number of M2 macrophages in lung tissues. In - vitro experiments have confirmed that ASC - Exos may reduce the recruitment of circulating monocyte - derived macrophages by inhibiting the expression of CCL7 in fibroblasts. Additionally, the highly enriched let - 7b and let - 7a in ASC - Exos may play a crucial role in inhibiting the expression of CCL7 in fibroblasts (Zhang et al., 2023a). The latest research shows that ASC - Exos may also alleviate the oxidative stress damage of lung fibroblasts by regulating mitochondrial function, thereby alleviating the process of pulmonary fibrosis (Li et al., 2021).

#### 2.3.5 Tumor immunotherapy

Immunotherapy has broad prospects in the field of tumor treatment. However, immunosuppression in the tumor microenvironment severely restricts its clinical application effectiveness. The team from the Institute of Special Wild Economic Animals and Plants, Chinese Academy of Agricultural Sciences (Yang et al., 2024) innovatively modified the exosomes derived from deer antler stem cells. By using an M2 macrophage - targeting peptide (M2Pep) for modification and encapsulating the Toll-like receptor 3 agonist poly (I:C), they successfully developed engineered deer antler stem cell exosomes (M2Pep - Exo (pIC)). With the targeting effect of M2Pep, M2Pep - Exo (pIC) can be highly enriched in tumor tissues. This engineered exosome can reprogram tumor - associated macrophages, promote the maturation of dendritic cells, increase the infiltration of cytotoxic T lymphocytes at the tumor site, and achieve effective antitumor immunotherapy. It is highly suppressive of primary tumor growth and metastasis whencombined with a PD - L1 antibody, providing new insights into combination immunotherapy. Recent developments indicate that metabolic reprogramming can be controlled in tumor cells sensitized to immunotherapy by ASC - Exos (Zhu et al., 2024).

#### 2.3.6 Treatment of neurodegenerative diseases

ASC-Exos have great neuroprotective and neural repair properties and indicate therapeutic efficacy in the preclinical models of Alzheimer’s and Parkinson’s diseases (AD and PD).

ASC-Exos reduced amyloid-β (Aβ) plaque formation by 40% and suppressed tau hyperphosphorylation in an AD mouse model, in conjunction with the improvement of spatial learning and memory functions (Yang et al., 2024). This neuroprotective activity is mediated by exosomal brain-derived neurotrophic factor (BDNF) and miR-132, which synergistically inhibit neuronal apoptosis and enhance Aβ clearance mechanisms.

ASC-Exos preserved dopaminergic cells in the substantia nigra pars compacta in an MPTP-induced PD model, and 35% of dopaminergic cells were found to be positive in tyrosine hydroxylase (TH). The deficit of motor coordination was also alleviated (Yang et al., 2024).

#### 2.3.7 Anti-aging and intervention in age-related diseases

ASC-Exos reverse aging-related phenotypes and enhance systemic physiologic activity and cross-species validation of preclinical models in rodents and non-human primates.

EVs_ABPC (ASC-Exos-derived extracellular vesicles) rejuvenated the epigenetic clock of aged mice by the equivalent of six to seven human years, 1.5-fold the bone density, and motor performance (Rotarod test) and cognitive (Y-maze test) (Hao et al., 2025).

Long-term delivery of EVs-ABPC decreased senescence markers (SA-β-gal, γ-H2AX) in several tissues (liver, kidney, and brain) in rhesus monkeys, attenuated age-related fibrosis, and had a favorable safety profile with no apparent adverse effects (Hao et al., 2025).

The potential translational applications include age-related sarcopenia, cardiovascular aging, and age-related macular degeneration, which could help address the global challenge of promoting healthy aging.

#### 2.3.8 Treatment of metabolic disorders

Preclinical studies have shown that ASC-Exos regulate glucose and lipid metabolism, thereby establishing a new therapeutic direction for the treatment of metabolic diseases.

Antler stem cell exosomes (ASC-Exos) improve metabolic outcomes in type 2 diabetes (T2D) mice, including enhanced insulin sensitivity and reduced fasting blood glucose. This is also due to exosomal miR-146a targeting IRAK1, a key mediator of insulin resistance; direct evidence shows that miR-146a-mediated IRAK1 inhibition alleviates diabetic metabolic dysfunction by modulating inflammatory signaling (Li et al., 2023). Liu et al. (2023) (Liu et al., 2023) also supported the metabolic regulatory ability of antler stem cell-derived exosomes, and the miR-146a-IRAK1 axis mechanism was proven in the exosome-based diabetic treatment study (Li et al., 2023).

ASC-Exos alleviated body weight and lipid profiles (triglycerides, cholesterol) in obese mice and reduced adipose tissue inflammation by 15% (Liu et al., 2023).

## 3 Discussion

Deer antler stem cell-derived exosomes (ASC-Exos) have emerged as a highly promising candidate in regenerative medicine, combining the regenerative potential of parent antler stem cells (ASCs) with the ability of exosomes to target the desired location. The technical underpinnings, functional pathways, and translational prospects of these systems have become more elucidated through a systematic review of existing study results, but significant limitations to clinical translation persist.

### 3.1 Technical progress and limitations

Three standard isolation techniques have formed the basis of ASC-Exos studies, with each having a different trade-off between yield, purity, and functional integrity. Ultracentrifugation, the gold standard, enables processing of large sample volumes and yields high total recoveries, making it suitable for preliminary functional screening and high-throughput cargo analysis (Thery et al., 2006). However, its laboriousness, the possibility of exosomal membrane damage, and the co-isolation of protein aggregates restrict its use in fine-scale mechanistic studies and in clinical-grade preparation (Corona et al., 2023; Zhang et al., 2023a). The size exclusion chromatography (SEC) maintains exosomes integrity and cargo stability through a mild separation mechanism resulting in high-purity vesicles with reproducible biological activity-a characteristic that makes it the best choice in preclinical efficacy studies and in studies that require a more detailed look at the mechanism (Lei et al., 2022; Mathivanan et al., 2010). Nevertheless, its poor scalability stems from low throughput and time-intensive operations, which constrain it to large-scale production. The highest purity is achieved by immunoaffinity capture, which targets specific surface markers (e.g., CD63, CD81) and enables functional isolation of a particular subpopulation to guide therapy (van Niel et al., 2018; Yang et al., 2024). However, the high cost, low yield, and potential changes in surface marker conformation are obstacles to its widespread implementation (Corona et al., 2023). These isolation strategies are supported by conventional identification procedures, such as morphological characterization by transmission electron microscopy (TEM), size distribution analysis by dynamic light scattering (DLS), and marker detection by Western blotting and flow cytometry, which is why ASC-Exos studies are reliable. The most notable impediment to clinical translation, however, remains the absence of uniform, large-scale production and purification guidelines.

Multi-dimensional biological effects of ASC-Exos are attributed to their encapsulated bioactive cargos (e.g., proteins, mRNAs, and miRNAs: let-7a/let-7b, miR-21-5p, and miR-146a-5p), which constitute a full-fledged regulatory network that participates in tissue repair and pathogenesis. There is steadily growing evidence that they stimulate the growth and motility of repair-associated cells (e.g., fibroblasts, vascular endothelial cells) via activation of signaling pathways such as the STAT3 and PI3K/Akt, e.g., miR-21-5p generated by ASC-Exos has been demonstrated to target PTEN to activate PI3K/Akt pathway, thus increasing fibroblast proliferation and leading to wound re-epithelialization (Meng et al., 2024; Wang et al., 2019; Zhang et al., 2023b). In terms of cell differentiation regulation, ASC-Exos precisely guide the lineage commitment of osteoblasts, chondrocytes, and neural stem cells: they upregulate osteogenic marker genes (Runx2, OCN) in mesenchymal stem cells to promote osteogenesis (Lei et al., 2022; Liu et al., 2020), induce chondrocyte differentiation by delivering Sox9 and Col2a1 mRNAs (Meng et al., 2024), and facilitate neural stem cell differentiation into mature neurons via transferring neurotrophic factors (BDNF, GDNF) and miR-124-3p (Li et al., 2014; Yang et al., 2024). Their immunomodulatory functions have been further refined by recent studies: beyond shifting macrophage polarization from M1 to M2 (via delivering miR-146a-5p to inhibit NF-κB signaling (Zhang et al., 2023a), ASC-Exos also suppress the proliferation of pro-inflammatory T cells (Th1, Th17) by downregulating IL-17 and IFN-γ secretion, promote regulatory T cell (Treg) expansion through upregulating Foxp3 expression, and inhibit dendritic cell maturation to reduce antigen presentation capacity—collectively reshaping the immune microenvironment toward a pro-repair state (Yang et al., 2024). Additionally, ASC-Exos exhibit anti-senescence effects by regulating DNA repair (e.g., upregulating BRCA1 expression) and senescence-related genes (p53, p21; (Lei et al., 2022), alleviate oxidative stress through enhancing the activity of antioxidant enzymes (SOD, CAT) and reducing reactive oxygen species (ROS) production (Li et al., 2021), and modulate metabolic reprogramming (e.g., shifting glycolysis to oxidative phosphorylation) in repair-competent cells to adapt to the hypoxic and nutrient-deficient repair microenvironment (Zhu et al., 2024). Despite these advances, the molecular mechanisms underlying ASC-Exos-mediated effects remain incompletely elucidated. For instance, the specific roles of individual cargos (e.g., miR-146a-5p, BDNF) in regulating metabolic reprogramming in tumors or metabolic disorders, the crosstalk between PI3K/Akt and NF-κB signaling pathways during tissue repair, and the precise mechanisms of target cell recognition (e.g., the role of exosomal surface proteins such as CD47) and cargo delivery require in-depth investigation (Liu et al., 2023; Zhu et al., 2024).

Multi-dimensional biological responses of ASC-Exos are based on encapsulated bioactive cargos (e.g., proteins, mRNAs, and miRNAs, including let-7a/7 and miR-21-5p), in a complete regulatory network of tissue repair and disease progression. They facilitate the growth and movement of cells involved in the repair process (e.g., fibroblasts, vascular endothelial cells) upon stimulating the signaling cascade, such as STAT3 and PI3K/Akt (Meng et al., 2024; Zhang et al., 2023b), and precisely regulate the differentiation of osteoblasts, chondrocytes, and neural stem cells via upregulating lineage-specific genes and delivering neurotrophic factors (Lei et al., 2022; Liu et al., 2020; Yang et al., 2024). Their effect on the immune microenvironment is successful by using immunomodulatory capabilities such as changes in macrophage polarization, preventing proliferation of pro-inflammatory T cells (Th1, Th17), promoting expansion of regulatory T cells (Treg), and preventing the maturation of dendritic cells (Yang et al., 2024; Zhang et al., 2023a). Additionally, ASC-Exos exhibit anti-senescence properties by regulating DNA repair and senescence-related genes, alleviating oxidative stress through increased activity of antioxidant enzymes, and responding to the repair microenvironment by modulating metabolic reprogramming (Lei et al., 2022; Li et al., 2021; Zhu et al., 2024). Despite this progress, the molecular processes underlying the effects of ASC-Exos remain incompletely understood. To provide an example, how individual cargos regulate metabolic reprogramming in tumors or metabolic diseases, the cross-interaction among various signaling pathways, the mechanisms of target cell recognition and cargo delivery need to be thoroughly examined (Liu et al., 2023; Zhu et al., 2024).

### 3.2 Translational potential and clinical challenges

ASC-Exos have shown significant potential in therapeutics in a wide range of conditions of clinical importance, overcoming the major bottlenecks of modern regenerative medicine-including inadequate efficacy of standard treatments, high immune rejection rates, and low biocompatibility. They are used in tissue repair, pro-angiogenic, anti-fibrotic, and collagen remodeling in the repair of bones with defects in the femur, in the inhibition of cartilage erosion in osteoarthritis, and in the acceleration of wound healing (including diabetic wounds) (Lei et al., 2022; Meng et al., 2024; Zhang et al., 2023b; Zuo et al., 2024). In disease treatment, ASC-Exos mitigate pulmonary fibrosis by inhibiting CCL7-mediated monocyte-macrophage recruitment and regulating mitochondrial function (Li et al., 2021; Zhang et al., 2023a); enhance tumor immunotherapy efficacy via engineered modification (e.g., M2Pep/poly (I:C)-loaded ASC-Exos) to reprogram the immunosuppressive tumor microenvironment (Yang et al., 2024); exert neuroprotective effects in Alzheimer’s and Parkinson’s disease models (Yang et al., 2024); reverse aging phenotypes in rodent and non-human primate models (Hao et al., 2025); and regulate glucose-lipid metabolism in type 2 diabetes and obesity (Liu et al., 2023). Nevertheless, the translational potential of ASC-Exos faces significant challenges. First, long-term safety and efficacy data are lacking: while preclinical models have demonstrated favorable biosafety profiles (Hao et al., 2025; Yang et al., 2024), clinical trials are required to evaluate potential long-term adverse effects, immunogenicity, and optimal dosage regimens. Second, the translational value of ASC-Exos in complex diseases (e.g., neurodegenerative disorders, age-related conditions) demands further validation through large-scale, multi-center preclinical studies. Third, specific delivery systems and combination approaches (e.g., using tissue-engineering scaffolds or precision medicine) need to be developed to enhance the specificity and efficacy of the therapeutic approach.

## 4 Conclusion

Overall, deer antler stem cell-derived exosomes (ASC-Exos) are a new therapeutic agent in regenerative medicine, with low immunogenicity and superior biocompatibility. They combine the natural regenerative benefits of deer horns with the enhanced efficiency of targeted exosome delivery, with strong clinical application potential. This review provides a systematic review of the existing body of research on ASC-Exos: technically, their multi-dimensional regulatory network through encapsulated bioactive cargos provides a reliable basis for their study and use; functionally, their wide-ranging therapeutic potential in terms of repairing tissues, treating diseases, anti-aging, and metabolic regulation may make them an all-purpose candidate in regenerative medicine. Future studies aim to develop ASC-Exos by pursuing three fundamental directions for the clinical translation of the technology. To begin with, rationalize large-scale production and purification methods to come up with practical, economical, and clinically consistent methods that would assess the balance between production, purity, and functional integrity-thereby fixing the present bottleneck of routinely industrialized methods. Second, elucidate the cargo-specific molecular pathways underlying exosome-mediated biological functions and the interplay of downstream signaling pathways, thereby providing a theoretical basis for precise ASC-Exos engineering and the design of specific therapeutic interventions. Third, conduct well-designed preclinical and clinical trials to confirm long-term safety and efficacy and to identify optimal administration schedules (e.g., dosage, route, frequency). Additionally, the combination of ASC-Exos with critical drug delivery systems (e.g., hydrogels, targeted peptides), tissue engineering systems (e.g., bioactive scaffolds), and precision medicine plans will improve therapeutic performance and applicability and increase their use in clinical practice.

Incorporating the latest advances in exosome research, ASC-Exos would be employed as a new treatment option in regenerative medicine to address incurable diseases and regenerate tissues. This will eventually help enhance global health. The review provides a comprehensive foundation for further research and clinical translation of ASC-Exos in regenerative medicine.
